# Distinct Lineages of *Schistocephalus* Parasites in Threespine and Ninespine Stickleback Hosts Revealed by DNA Sequence Analysis

**DOI:** 10.1371/journal.pone.0022505

**Published:** 2011-07-19

**Authors:** Nicole Nishimura, David C. Heins, Ryan O. Andersen, Iain Barber, William A. Cresko

**Affiliations:** 1 Institute of Ecology and Evolution, University of Oregon, Eugene, Oregon, United States of America; 2 Department of Ecology and Evolutionary Biology, Tulane University, New Orleans, Louisiana, United States of America; 3 Institute of Neuroscience, University of Oregon, Eugene, Oregon, United States of America; 4 Department of Biology, College of Medicine, Biological Sciences and Psychology, University of Leicester, Leicester, United Kingdom; Institute of Marine Research, Norway

## Abstract

Parasitic interactions are often part of complex networks of interspecific relationships that have evolved in biological communities. Despite many years of work on the evolution of parasitism, the likelihood that sister taxa of parasites can co-evolve with their hosts to specifically infect two related lineages, even when those hosts occur sympatrically, is still unclear. Furthermore, when these specific interactions occur, the molecular and physiological basis of this specificity is still largely unknown. The presence of these specific parasitic relationships can now be tested using molecular markers such as DNA sequence variation. Here we test for specific parasitic relationships in an emerging host-parasite model, the stickleback-*Schistocephalus* system. Threespine and ninespine stickleback fish are intermediate hosts for *Schistocephalus* cestode parasites that are phenotypically very similar and have nearly identical life cycles through plankton, stickleback, and avian hosts. We analyzed over 2000 base pairs of COX1 and NADH1 mitochondrial DNA sequences in 48 *Schistocephalus* individuals collected from threespine and ninespine stickleback hosts from disparate geographic regions distributed across the Northern Hemisphere. Our data strongly support the presence of two distinct clades of *Schistocephalus*, each of which exclusively infects either threespine or ninespine stickleback. These clades most likely represent different species that diverged soon after the speciation of their stickleback hosts. In addition, genetic structuring exists among *Schistocephalus* taken from threespine stickleback hosts from Alaska, Oregon and Wales, although it is much less than the divergence between hosts. Our findings emphasize that biological communities may be even more complex than they first appear, and beg the question of what are the ecological, physiological, and genetic factors that maintain the specificity of the *Schistocephalus* parasites and their stickleback hosts.

## Introduction

Although the processes of competition and predation have historically received the lion's share of focus in evolutionary ecology research, the importance of parasitism as an evolutionary force – and its potential in structuring community dynamics – is being increasingly recognized [1]–[5]. Parasites can have far-reaching and often unexpected effects on biological communities [6]. One major way in which parasites can influence food webs, for example, is by influencing the probability of survival of host organisms through alterations of their antipredator behavior [7]. Such behavioral changes are potentially adaptive where parasite transmission relies on one host being consumed by the next [8]–[12]. Through their myriad effects on host organisms, parasites can also alter the competitive ability, growth, sexual maturation, sexual attractiveness and parental ability of host organisms, and hence have considerable fitness implications for hosts. Understanding parasitic interactions in the wild is very important for accurately describing community complexity [6].

Despite the importance of parasitic interactions, we are just beginning to understand the evolutionary origins of these complex parasite systems and their impacts on multiple levels of communities [5], [7], [13]–[16]. A number of basic questions remain unanswered. For example, how often do closely related and geographically overlapping host species share phenotypically similar parasites? How often have these parasites co-speciated with their hosts to form reciprocally specific, yet cryptic, host-parasite interactions [17]? What traits have evolved in both the hosts and parasites to produce the specificity of these interactions, and what are the genetic, physiological and developmental systems that are changing in both the hosts and parasites as they co-evolve? Furthermore, when specific interactions evolve in closely related pairs of hosts and parasite species in parallel, are the genetic bases of these phenotypic changes also parallel? Finally, once evolved, how do these compartmentalized parasitic interactions affect the structure of communities?

These questions can be addressed by studying natural systems of closely related host species that live in both sympatry and allopatry, and either share a parasite or exhibit reciprocal specificity. An excellent system with which to address these questions is the fish family Gasterosteidae and their cestode parasites [18], [19]. The threespine stickleback (*Gasterosteus aculeatus*) has long been a model for studying evolutionary processes and ecological interactions [20]–[26], and more recently has become an important focus for studies of the genetic basis of evolution in the wild [26]–[33]. Oceanic threespine stickleback have repeatedly given rise to derived freshwater populations that have evolved along many phenotypic axes [34], [35], including the ability to cope with a large number of parasites that differ according to the habitat in which they are found [36]. The ninespine stickleback (*Pungitius pungitius*) is well supported as the sister lineage to threespine stickleback, and is also being developed into a model for evolutionary and ecological studies [37]–[42]. Ninespine stickleback also has a widespread, circumarctic distribution that frequently overlaps with threespine stickleback, and the two species can co-occur in the same lakes and rivers [21], [43]. In these locations, similarities between the parasite communities of both hosts have been documented [36], [44].

One of the best-studied parasites of stickleback is the widespread, complex diphyllobothriidean cestode parasite, *Schistocephalus solidus*, which infects threespine stickleback [45]. This species has become an important model for studies of the ecology and evolution of relationships in parasitology [19], [46], [47]. The parasite begins life as a briefly free-swimming coracidium, which must quickly be eaten by one of several species of copepod in order to develop into a procercoid. After as little as 8 days of development within the copepod host, the procercoid becomes infective and can be transmitted via predation to the stickleback host. In stickleback *S. solidus* undergoes a period of explosive growth as a plerocercoid that can increase 4000 times in size in less than six weeks [48]–[50], filling a large proportion of the coelom of the fish. *S. solidus* fuels its growth by stealing nutrients from the host stickleback. The fish and parasite are eaten by any of about 40 piscivore avian species. In the intestines of the bird the worm becomes sexually mature and distributes coracidia to any number of other lakes via eggs that pass out with the feces of the terminal avian host [51]–[54].

Although most of the work on *Schistocephalus* has been performed in the threespine stickleback host, a *Schistocephalus* parasite with a remarkably similar morphology and life cycle infects ninespine stickleback [55]–[57]. This phenotypic similarity led to the hypothesis that the two fish are each infected with a single *Schistocephalus* species that must somehow have evolved to cope with the demands of two different hosts. This ‘shared parasite’ hypothesis is supported by the fact that many species within Diphyllobothriidae are apparently generalists when it comes to their hosts [58], [59] and are able to infect a number of different species at all stages of their life cycle. In addition, the ecological niches of threespine and ninespine stickleback overlap, and the two stickleback species co-occur beyond expectation on both the micro- & macro habitat scales and in overall diet [60]. Dartnall [55] surveyed both threespine and ninespine stickleback parasite fauna and concluded that there were few differences between the parasites that affect the two fish hosts. Therefore, since the non-stickleback life stages of *S. solidus* infect copepods and birds that are both the prey source and predators respectively of both types of stickleback, and since threespine and ninespine niches overlap, the separation of *Schistocephalus* between stickleback hosts seemed difficult to achieve.

Despite the phenotypic and life history similarities between the *Schistocephalus* parasites infecting the two stickleback hosts, an alternative hypothesis is that these parasites are actually two cryptic species, *S. solidus* and *S. pungitii*, that have been independently co-evolving to threespine and ninespine stickleback hosts respectively. Several lines of evidence support this hypothesis. Dubinina [45] described the *S. pungitii* species and distinguished between it and *S. solidus* by slight differences in numbers of proglottids, a traditional taxonomically important morphological characteristic in cestodes. To test Dubinina's taxonomic distinction, Bråten [61] both surgically injected procercoids and transplanted plerocercoids from threespine stickleback hosts into the body cavities of several co-occurring fish hosts, only to see them quickly die. Similarly, Orr et al [62] found that *S. solidus* plerocercoids slowed their growth and died within two weeks after a ninespine host ingested an infected copepod. Furthermore, recent morphological and molecular studies of *Schistocephalus* plerocercoids recovered from sculpins (*Cottus gobio*) in Icelandic rivers showed significant deviation in microsatellites from plerocercoids recovered from local threespine sticklebacks, providing further evidence that cryptic speciation may be widespread in the genus [63]. Lastly, molecular studies have uncovered considerable genetic diversity in a closely related and cosmopolitan species, *Ligula intestinalis*, suggesting the existence of cryptic lineages [64]–[67].

We decided to test two hypotheses for the relationships of *Schistocephalus* spp. and their stickleback hosts – a single shared parasite or cryptic species – using molecular genetic data. Specifically we examined geographic variation in two mitochondrial DNA (mtDNA) regions, the NADH1 and COX1 genes. We were able to collect representative threespine stickleback from sites across the Northern Hemisphere (Alaska, Oregon and Wales), and ninespine stickleback from part of this range, from which we extracted *Schistocephalus* parasites for genetic analysis. If the *Schistocephalus* parasites are a single lineage shared between the two stickleback hosts, all phylogeographic clustering will be consistent with isolation by distance. Alternatively, if the ‘two cryptic species’ hypothesis is true, then the main determinant of genetic structuring will primarily be the host species from which the *Schistocephalus* were collected, with isolation by distance present only within the clades of parasites drawn from the same species. Here we present molecular genetic data from *Schistocephalus* species from multiple different host individuals, populations and the two stickleback species that clearly support the cryptic species hypothesis.

## Results

### Cloning and sequencing of novel Schistocephalus mtDNA sequence

We successfully obtained mtDNA sequence data from 48 *Schistocephalus* samples from the two host species and three geographic regions (Table 1 and Table 2). We were able to generate the entire sequence of the COX1 and NADH1 mitochondrial genes from the parasites of each host via degenerate PCR, cloning and sequencing. These archetypal sequences, from parasites taken from the two host stickleback species provided the first indication of extensive genetic diversity. To examine whether this diversity is partitioned among species and populations in a meaningful way, we developed new specific PCR primers for worms from each of the hosts and generated sequence data for each of the 48 samples. Not all PCR primer pairs generated the same robust amplification in each species, but we were able to find at least one pair that produced amplification in each of the species. Subsequent TA cloning and sequencing generated over 2500 bp of mitochondrial sequence from the majority of the 48 individual worms from the two host species, three geographic regions and nine populations. For all subsequent sequence variation and phylogenetic measures we used the largest sequence region of each gene that was successfully analyzed in each sample, yielding data for 1390 bp of COX1 and 809 bp of NADH1, or a concatenated total sequence length of 2199 bp for each of the samples. Since these genes are both part of the non-recombining mitochondrial genome, we concatenated the sequence and examined patterns of diversity among the parasites with respect to host species, geographic region and population. These represent the largest set of population-level genetic data yet generated for *Schistocephalus* species.

**Table 1:** Collection locations and stickleback hosts for *Schistocephalus* samples.

Population Name	Abbreviation	Global Region	Latitude and Longitude	Stickleback Host
Mud Lake	MDL	Alaska, USA	N61.5617; W148.9505	*G. aculeatus* & *P. pungitius*
Dog Bone Lake	DBL	Alaska, USA	N60.6958; W151.2875	*P. pungitius*
Falk Lake	FAL	Alaska, USA	N61.5658; W149.0486	*G. aculeatus*
Seymour Lake	SEL	Alaska, USA	N61.6111; W149.6653	*G. aculeatus*
Scout Lake	SCL	Alaska, USA	N60.5331; W150.8418	*G. aculeatus*
South Twin Lake	STL	Oregon, USA	N43.7125; W121.7652	*G. aculeatus*
Pony Creek Reservoir	PCR	Oregon, USA	N43.3655; W124.2634	*G. aculeatus*
Pond-yr-Oerfa	PYO	Wales, UK	N52.3610; W3.8794	*G. aculeatus*
Llyn Frongoch	LLF	Wales, UK	N52.4008 W3.8703	*G. aculeatus*

**Table 2:** Haplotype distributions among populations, regions and host species.

Haplotype	# of Sequences	Locations	Regions	Host Species
AK_01	1	Mud Lake	Alaska, USA	*G. aculeatus*
AK_02	1	Mud Lake	Alaska, USA	*G. aculeatus*
AK_03	1	Mud Lake	Alaska, USA	*G. aculeatus*
AK_04	1	Falk Lake	Alaska, USA	*G. aculeatus*
AK_05	2	Falk Lake	Alaska, USA	*G. aculeatus*
AK_06	1	Falk Lake	Alaska, USA	*G. aculeatus*
AK_07	2	Falk Lake & Scout Lake	Alaska, USA	*G. aculeatus*
AK_08	1	Falk Lake	Alaska, USA	*G. aculeatus*
AK_09	1	Falk Lake	Alaska, USA	*G. aculeatus*
AK_10	1	Falk Lake	Alaska, USA	*G. aculeatus*
AK_11	1	Seymour Lake	Alaska, USA	*G. aculeatus*
AK_12	1	Seymour Lake	Alaska, USA	*G. aculeatus*
AK_13	1	Seymour Lake	Alaska, USA	*G. aculeatus*
AK_14	2	Seymour Lake & Scout Lake	Alaska, USA	*G. aculeatus*
AK_15	1	Scout Lake	Alaska, USA	*G. aculeatus*
AK_16	1	Scout Lake	Alaska, USA	*G. aculeatus*
AK_17	1	Scout Lake	Alaska, USA	*G. aculeatus*
AK_18	1	Scout Lake	Alaska, USA	*G. aculeatus*
AK_19	1	Scout Lake	Alaska, USA	*G. aculeatus*
AK_20	1	Scout Lake	Alaska, USA	*G. aculeatus*
AK_21	1	Mud Lake	Alaska, USA	*P. pungitius*
AK_22	1	Mud Lake	Alaska, USA	*P. pungitius*
AK_23	1	Mud Lake	Alaska, USA	*P. pungitius*
AK_24	3	Mud Lake & Dog Bone Lake	Alaska, USA	*P. pungitius*
AK_25	1	Mud Lake	Alaska, USA	*P. pungitius*
AK_26	2	Mud Lake	Alaska, USA	*P. pungitius*
AK_27	1	Mud Lake	Alaska, USA	*P. pungitius*
AK_28	1	Dog Bone Lake	Alaska, USA	*P. pungitius*
OR_29	3	South Twin Lake	Oregon, USA	*G. aculeatus*
OR_30	2	South Twin Lake	Oregon, USA	*G. aculeatus*
OR_31	1	Pony Creek Reservoir	Oregon, USA	*G. aculeatus*
OR_32	2	Pony Creek Reservoir	Oregon, USA	*G. aculeatus*
CY_33	2	Pond-yr-Oerfa& Llyn Frongoch	Wales, UK	*G. aculeatus*
CY_34	3	Pond-yr-Oerfa& Llyn Frongoch	Wales, UK	*G. aculeatus*
CY_35	1	Llyn Frongoch	Wales, UK	*G. aculeatus*

### Nucleotide sequence variation and partitioning of molecular variance

The sequences were surprisingly diverse. The 48 individual sequences were mostly different, condensing only slightly to 35 unique haplotype sequences (Table 2). The genetic diversity within each population was moderate and similar to studies in other organisms, with π ranging from approximately 0.001 to 0.006 (Table 3). The first indication that a significant proportion of genetic diversity is partitioned among *Schistocephalus* hosts arises from examining the pairwise genetic diversity between populations (Table 4). Comparisons between pairs of populations within the two stickleback hosts and regions exhibited genetic variation that was not very different than the diversities within populations. Comparisons among threespine hosts from different regions showed significantly more diversity. By far the greatest diversity was observed in comparisons among sequences from threespine and ninespine hosts, nearly two orders of magnitude larger than the differences among even distantly separated populations within hosts. Strikingly, this large difference existed even between *Schistocephalus* when those sequences were drawn from worms collected from the different hosts living in the same Alaskan lakes (Table 4).

**Table 3:** Genetic diversity (+/−1 S.E.) within *Schistocephalus* populations from each host.

Population	Host	Average pairwise differences	Π
Mud Lake	*P. pungitius*	4.96+/−2.67	0.0023+/−0.0014
Dog Bone Lake	*P. pungitius*	3.33+/−2.27	0.0015+/−0.0013
Mud Lake	*G. aculeatus*	7.00+/−5.11	0.0033+/−0.0034
Falk Lake	*G. aculeatus*	5.36+/−2.86	0.0026+/−0.0016
Seymour Lake	*G. aculeatus*	10.33+/−5.88	0.0048+/−0.0032
Scout Lake	*G. aculeatus*	6.67+/−3.54	0.0033+/−0.0020
South Twin Lake	*G. aculeatus*	3.00+/−1.85	0.0014+/−0.0010
Pony Creek Reservoir	*G. aculeatus*	12.00+/−7.33	0.0058+/−0.0044
Pond-yr-Oerfa	*G. aculeatus*	10.67+/−6.55	0.0049+/−0.0037
Llyn Frongoch	*G. aculeatus*	10.67+/−6.55	0.0049+/−0.0037

**Table 4:** Genetic divergence among host species, regions, and populations in terms of the average number of pairwise nucleotide differences between haplotypes in the pooled sample from both populations, with host stickleback species and region represented along the top and left columns.

	*P. pungitius*		*G. aculeatus*						
	Alaska		Alaska				Oregon		Wales
	MDL	DBL	MDL	FAL	SEL	SCL	STL	PCR	PYO
DBL	5.01								
MDL	213.52	215.00							
FAL	219.64	220.88	0.34						
SEL	217.98	219.75	0.71	1.36					
SCL	217.46	218.76	0.10	0.26	0.13				
STL	214.47	215.73	23.50	26.74	26.23	25.48			
PCR	210.77	212.39	18.83	21.88	21.33	20.45	9.47		
PYO	229.14	231.33	78.50	80.80	81.42	80.52	92.60	87.17	
LLF	229.48	231.67	76.83	79.09	80.08	78.95	91.60	84.94	2.11

Similarly, the Analysis of Molecular Variance (AMOVA) results clearly show very significant structuring among stickleback hosts and regions, but relatively little among populations within regions (Tables 5 and 6). Most significantly, when all of the sequence data from Schistocephalus from both hosts were included in a single analysis, nearly 84% of the total variation was partitioned among the worms from the two different host species (Table 5), with only a very small proportion (∼14%) partitioned across populations distributed across the Northern Hemisphere. When the Schistocephalus mtDNA sequence data only from threespine hosts were analyzed (Table 6), this reduced set of variation became partitioned primarily among regions (∼87% of the variation across Alaska, Oregon and Wales), with only approximately ∼1% of the variation partitioned across populations within regions. The remaining 12% of the variation partitioned across individuals within populations. Clearly, the most significant factor determining the partitioning of genetic variation is the host stickleback species. Geographic region within host is less important but still significant, while partitioning among populations within regions is negligible.

**Table 5:** Analysis of Molecular Variance (AMOVA) grouping populations when *Schistocephalus* sequences are grouped into whether the parasite came from a threespine or ninespine host.

Source of variation	d.f.	Sum of squares	Variance components	Percentage of variation	p-value
Among groups	1	1709.740	96.02661	83.51	0.01760
Among populations within groups	8	560.884	15.69276	13.65	0.00000
Within populations	36	117.485	3.26346	2.84	0.00000
**Total**	45	2388.109	114.98283		

**Table 6:** Schistocephalus mtDNA sequence data only from threespine hosts.

Source of variation	d.f.	Sum of squares	Variance components	Percentage of variation	p-value
Among groups	2	458.695	23.00264	86.63	0.00684
Among populations within groups	5	23.114	0.32634	1.23	0.10166
Within populations	27	87.076	3.22504	12.15	0.00000
**Total**	34	568.886	26.55402		

### Phylogenetic patterns

**Figure 1:**
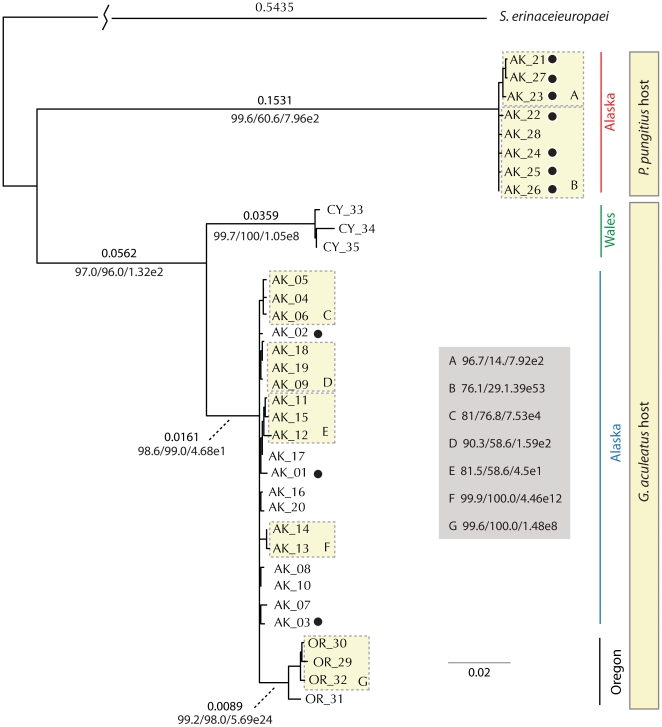
A reconstruction of the phylogenetic relationship of the 35 concatenated COX1 and NADH1 haplotype sequences from *Schistocephalus* collected from two different stickleback hosts and across three geographic regions. The tree is rooted using *Spirometra erinaceieuropaei* sequence as an outgroup, which is the closest cestode relative for which there was sufficient mtDNA sequence. The tree was reconstructed using Parsimony, Maximum Likelihood, and Bayesian approaches, and all gave qualitatively similar results (Maximum Likelihood topology is shown). In particular, the deepest division is between sequences from *P. pungitius* hosts (top) and *G. aculeatus* host (bottom), with these sequences being different, on average, at nearly 20% of the sites in the sequence (substitution rate above branch). This division is highly supported regardless of being measured by Bayesian Posterior Probability, Bootstrap, or Likelihood Ratio (support below branch). The next supported division is between *Schistocephalus* from Wales as compared to Oregon and Alaska, but with a sequence divergence less than 5%. The final strongly supported division are *Schistocephalus* from Oregon as compared to Alaska, but with a sequence divergence of less than 1%. Black dots represented *Schistocephalus* samples collected from either threespine or ninespine hosts from within Mud Lake, Alaska.

Both the Maximum Likelihood and Bayesian methodologies provided the same topology for the unique DNA sequences. Two major clades exist that are separated by approximately 20% of the sequence variation. These two clades are highly supported, and correspond precisely with the two different stickleback hosts from which the schistocephalus were drawn (Figure 1). Importantly, even *Schistocephalus* sequences from the same lake were partitioned between the two clades if the parasites came from different stickleback hosts (Figure 1) such that *Schistocephalus* from globally distributed threespine stickleback are all more closely related to one another than parasites collected from threespine and ninespine hosts in the same Alaskan lake.

A second level of significant divergence exists within the clade of worms from the threespine fish. Worms from Alaska, Oregon and Wales each cluster with one another by region, and these clusters are highly supported (Figure 1). The clustering fits a pattern of isolation by distance, and as expected the regions from the Pacific basin cluster to the exclusion of the Wales population from the Atlantic Basin. The difference between Wales and the Oregon/Alaska clade is only approximately 5%, whereas the difference between the Oregon and Alaska clades is less than 1% sequence differentiation (Figure 1). In contrast to the two levels of host species and major geographic regions, little significant partitioning was observed among populations or haplotypes within regions, supporting a role for gene flow mediated by the movement of birds between lakes within a region.

### Amino acid substitutions among host species and geographic regions

**Figure 2:**
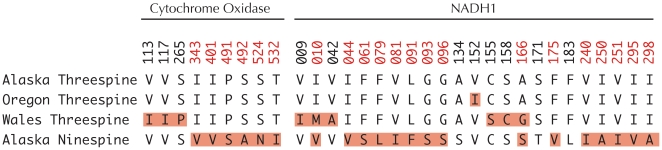
An alignment of the translated protein sequences *Schistocephalus* from Alaskan, Oregon and Wales threespine hosts, as well as *Schistocephalus* from ninespine hosts. Only positions that are variable in at least one comparison are represented in this alignment. Using the Alaskan threespine host sequence as a reference, only a single AA difference (red highlighting) occurs at position 152 of NADH1 from a Valine to Isoleucine in the Oregon threespine hosts. Nine differences occur between *Schistocephalus* collected from threespine hosts in the Pacific vs. Atlantic basins. In comparisons between *Schistocephalus* collected from threespine and ninespine hosts in Alaska twenty one residue changes exist (position numbers in red), many of which are likely to change the structure of the proteins.

Based upon the fact that the majority of phylogenetic structuring of the sequences was among host species and large geographic regions, we generated translations from the consensus sequences from both three and ninespine hosts from Alaska, Oregon and Wales. Identifying these protein coding changes provides an indication of how many of the nucleotide changes are potentially functional and subject to natural selection. These translations showed that the majority of SNPs segregating among the threespine populations were in silent sites, leading to very similar protein sequences between worms drawn from threespine hosts from Alaska and Oregon. Over this geographic scale only one nonsynonymous change was observed in residue 152 of NADH1 (Figure 2). In comparison, the differences between worms from threespine hosts from the Pacific vs. Atlantic Ocean basins exhibit greater divergence, with nine residue changing substitutions (Figure 2). In contrast, a very different pattern is observed when comparing the sequences from ninespine hosts to those from threespine hosts. A total of twenty one residues exhibit fixed differences between worms drawn from the two hosts (Figure 2). These 21 amino acid differences between *Schistocephalus* sequences from different hosts exists even between worms drawn from the two stickleback species living in the same lake in Alaska. These data further support the hypothesis that the nature of the genetic variation among *Schistocephalus* from the same and different host is qualitatively different, and likely represents two different *Schistocephalus* species that are specialized to use either threespine or ninespine stickleback.

## Discussion

The extent to which parasites are locally adapted to host races or species in natural populations remains largely unknown. We have shown that the relationships between *Schistocephalus* parasites and stickleback intermediate hosts can be quite specific, even when those species occupy similar niches in the same geographic locale and even the same lake. The pattern of partitioning of mtDNA genetic data provides strong evidence for the presence of two different lineages of *Schistocephalus*, one that exclusively infects threespine stickleback and the other that infects ninespine. These distinct lineages may represent separate species, the previously described *S. solidus* and *S. pungitii* (Dubinina, 1959). This conclusion is supported by our data because for worms from the threespine host there appears to be significant gene flow among even fairly geographically distant populations within a region, such as the populations in the geographically isolated MatSu and Kenai regions of Alaska, or even between geographically distinct regions such as Oregon and Alaska. In sharp contrast, *Schistocephalus* from ninespine stickleback are very differentiated from those extracted from threespine stickleback, even in lakes where these host species co-occur. The degree of divergence (∼20%) is so deep between the *S. solidus* and *S. pungitius* lineages that the speciation of the parasites most likely occurred shortly after the speciation of the stickleback hosts approximately 20–25 mybp [68]–[70].

One of the first conclusions from our work is that phenotypically similar species are infecting different but related hosts in a cryptic parasitic relationship. The use of molecular tools allows a definitive test of this hypothesis. Our data show that cryptic relationships among parasites and hosts can be present even in species that have been the focus of extensive previous work, and therefore these types of cryptic relationships may be much more common in nature than is presently appreciated. In systems less well studied than stickleback, for which fewer genetic tools are available, the opportunity may not yet exist for testing hypotheses of cryptic parasitic relationships. To examine fully this assertion will require more extensive molecular population genetic and phylogenetic studies of related species and lineages that are infected by phenotypically similar parasites. If cryptic parasitic relationships are more common in nature, as we hypothesize, this will have significant impacts on our understanding of the complexity of communities in the wild.

In addition to the cryptic parasitic relationship between stickleback species hosts, a further pattern that emerges from our data is the deep phylogeographic patterning within the *Schistocephalus solidus* lineage that infects threespine stickleback. A significant amount of differentiation exists between worms drawn from threespine hosts in the Atlantic and Pacific basins. Particularly notable is that although only a single amino acid substitution occurs between *Schistocephalus solidus* from Oregon and Alaskan threespine stickleback hosts, these Pacific Basin worms differ by nine substitutions from worms from Wales in the Atlantic Ocean basin. The possibility therefore exists of even more cryptic parasitic relationships within the clade of *Schistocephalus* that infects different threespine populations across the Northern Hemisphere. A much more detailed and in depth global phylogeographic study of *Schistocephalus solidus* is warranted to test this hypothesis.

Now that these cryptic parasitic relationships have been identified, a key problem is to understand the mechanistic basis for establishing and maintaining these specific relationships among *Schistocephalus* species and their stickleback hosts. What are the ecological contexts by which the specific relationships can be maintained? Stickleback and copepods are intermediate hosts for *Schistocephalus*, with sexual reproduction occurring in the primary avian hosts. One hypothesis for the specificity of the *Schistocephalus* in the stickleback hosts is that they are also specialized to different plankton and bird hosts, producing two independent, mutually exclusive cycles in which the two stickleback species are not exposed to the alternate parasites. For this ‘ecological separation’ hypothesis to be correct, prey choice by both the stickleback species on copepods, and bird prey choice on stickleback, must be very specific and partitioned. This hypothesis could be tested using the molecular tools we have already developed for this project by collecting individual plankton and bird gut contents or fecal material and asking whether unique lineages of *S. solidus* and *S. pungitius* mtDNA are present in different copepod and avian lineages.

An alternative hypothesis is that cross-exposure of *G. aculeatus* and *P. pungitius* to *S. solidus* and *S. pungitii* occurs regularly, but because of co-evolution between the stickleback hosts and *Schistocephalus* parasites, infections only establish when each *Schistocephalus* species infects the correct host. The prey choice of copepods by stickleback, or avian predation on stickleback, may not be so precise as to ensure that two completely separable cycles exist. Instead, threespine stickleback may be preying on copepods that are infected with both *S. solidus* and *S. pungitius* parasites, but only the *S. solidus* are able to become established in the threespine stickleback host. If this is true, then the prediction is that *S. solidus* and *S. pungitius* have evolved ways to circumvent the threespine stickleback and ninespine stickleback defenses respectively, but in doing so have lost the ability to infect the alternative host [71]–[74]. This result is not unprecedented, and is similar to work showing that closely related species of mice with overlapping ranges still have quite distinct immune systems and responses to parasites [75]–[78]. Furthermore, we do not present these ecological and immune system hypotheses as the only, or even the most likely, hypotheses, and use them only as examples of the types of ecological questions that can now be addressed with this system based upon our findings.

The fact that these two lineages are strongly separated, even between hosts that co-exist in the same lake, argues strongly for antagonistic trade-offs during the co-evolutionary process making the ability to infect both hosts impossible. Evolving the ability to cope with the defenses of one host probably reduces the probability of efficiently doing so with the other. The previous work by Dubinina [45], showing that heterospecific *Schistocephalus* species are rejected when transplanted into alternative hosts, fits this hypothesis. This ‘antagonistic co-evolution’ hypothesis can also be tested with the tools we developed for this project by sampling *Schistocephalus* mtDNA lineages from both copepods and bird hosts. If the two lineages are co-mingled in either one or both of the hosts, this would argue for the specific rejection of heterospecific parasites during stickleback infection.

A particularly interesting unanswered question is how the *Schistocephalus* parasite is able to almost completely control the morphology, physiology and behavior of the stickleback host during a time period when the fish is most likely able to be eaten by birds [19], [79]. *Schistocephalus* infection changes both the morphology and behavior of the stickleback host in ways that are likely to increase the probability of transmission. The parasite also modifies the behavior of the fish, causing it to swim slowly near the surface of the water during the day, and to have dramatically reduced escape responses to predator stimuli [80]–[83]. In some Alaskan populations, infected fish even become de-melanised, rendering them highly visible to avian predators that attack from above [84]. The developmental, physiological and genetic basis of the ability of the *Schistocephalus* parasite to co-opt the host's soma is largely unknown, though there is evidence that modulation of brain monoamines may be involved [85].

In summary, we used molecular genetic tools to demonstrate clearly the presence of specific relationships between two cryptic species of *Schistocephalus*, *S. solidus* and *S. pungitius,* with threespine and ninespine stickleback hosts respectively. Despite this parasitic relationship being a very well studied ecological system in the wild, this is the first definitive test of this cryptic co-evolutionary relationship. Our data support the view that cryptic parasitic relationships may be more common in nature than we now know e.g., [17], [86]–[88], and that parasitism may be a much more ubiquitous and powerful interspecific interaction structuring communities in the wild than is presently appreciated.

## Materials and Methods

### Ethics statement

All collections of stickleback fish were made under the auspices of relevant state, national and international permits, and were performed according to approved institutional animal care and welfare protocols.

### Sample origin and extraction

We collected *Schistocephalus* infected threespine and ninespine stickleback from three globally disparate locations (Table 1). In both Wales and Oregon, replicate infected threespine stickleback were collected from two distinct populations. In Alaska, we collected replicate infected samples from five populations. These five populations were spilt into two regions of lakes. Three separate lakes clustered in the Matanuska-Susitna Borough of south-central Alaska and two separate lakes clustered on the northern Kenai Peninsula [28]. These lake regions are approximately 150 linear km from one another. Infected ninespine stickleback were collected from a single lake in each of these regions. Mud Lake, in the Matanuska-Susitna Borough, contained both threespine and ninespine stickleback, whereas only ninespine stickleback were present in Dog Bone Lake on the Kenai Peninsula. Stickleback collections were made using minnow traps or dipnets. *Schistocephalus* samples were selected such that each individual plerocercoid came from a different fish to avoid potential bias of related *Schistocephalus* infections of a single host. In cases of multiple infections, the largest plerocercoid was analyzed. These samples were immediately preserved in 100% ETOH in the field. DNA was later extracted from whole tissue samples using Epicentre's MasterPure Complete DNA and RNA Purification Kit (Epicentre Cat. # MC85200).

### Generating novel Schistocephalus mtDNA sequences

Only small fragments of mtDNA had previously been sequenced from *Schistocephalus*. We therefore used degenerate PCR to clone and sequence additional, larger fragments of the complete COX1 and NADH1 genes from *Schistocephalus*. To design degenerate primers we created an alignment of large fragments of the mitochondrial genome of related Eucestode species (*Hymenolepis diminuta*, *Taenia saginata*, *Taenia asiatica*, *Diphyllobothrium latum*, *Diphyllobothrium nihonkaiense*) with the fragments of *S. solidus* sequence available in GenBank. Species were chosen based on availability of published mitochondrial genomes at the time. These fragments included the entire target gene coding region and significant portions of the upstream and downstream sequences, approximately 2300 bp for COX1 and 1900 bp for NADH1. Multiple combinations of degenerate primers were designed by hand to cover the entire coding region with a preference for placing primers outside of coding regions but within relatively conserved regions across the species alignment. PCR amplification was performed on *Schistocephalus* samples from both threespine and ninespine to find a primer pair that produced products of approximately the correct size and the minimal number of extraneous bands (Supp. Table 1). Fragments of the correct size were isolated and cloned into the TOPO TA vector (Invitrogen Cat. # K4530-20) using standard protocols. Clones were isolated and screened for inserts of the correct size, which were then Sanger sequenced in the University of Oregon Genomics Facility.

### Bioinformatics of novel sequences and specific primer design

The resulting sequences from the degenerate PCR were correctly oriented and assembled using the computer program Geneious. Coding region coverage from both host species was confirmed using an alignment of the two most closely related species, *D. latum* and *D. nihonkaiense*. From these aligned sequences we designed sets of stickleback host specific primers, again positioning primers to maximize coding region coverage and to place them within conserved regions across *Schistocephalus* sequences from both threespine and ninespine hosts. Because of the sequence divergence among *Schistocephalus* from the different locations not all specific primer pairs worked in each sample. From the numerous different primer pair combinations we were able to amplify a minimum overlapping region of approximately 870 bp region of NADH1 (Supp. Table 1) and ∼1480 bp region of COX1 for *Schistocephalus* from both host types and from all regions. We amplified these fragments from each sample, and purified the PCR products using Zymo DNA Clean and Concentrator (Cat #D4004). To ensure best quality sequence return, we used different internal primers for direct Sanger sequencing. In a few cases where direct sequencing of PCR products proved difficult, we TopoTA cloned the fragments and sequenced them from the vector as described previously for the sequences generated via degenerate PCR.

### Sequence alignments, population genetic and phylogenetic analyses

Sequences were aligned using MUSCLE [89] in Geneious with the default parameters. To account for the heterogeneity in fragment length mentioned above, we trimmed all sequences to the shortest common length of 1390 bp for COX1 and 809 bp for NADH1, and concatenated these to a total length of 2199 bp. As an outgroup we included the homologous sequence from *Spirometra erinaceieuropaei*, which was determined to be the closest relative of the *Schistocephalus* clade for which appropriate mtDNA was available. Molecular population genetic diversity indices, such as the average number and frequency of pairwise differences within and among populations, were calculated using the software Arlequin [90] using the full set of sequences. In addition, partitioning of the distribution of molecular variation was determined using AMOVA framework also implemented in Arlequin. Two nested structures were tested. All populations were first tested within higher groups of threespine and ninespine hosts, and subsequently ninespine host sequences were removed and the remaining *Schistocephalus* sequences from only threespine hosts were grouped by the regions from which they were collected; Alaska, Oregon and Wales.

The 48 concatenated sequences condensed to 35 unique haplotypes (Table 2), and we used these for phylogenetic analyses. In order to determine the best model for the Maximum Likelihood and Bayesian phylogenetic analyses of the nucleotide sequences, we used the program jModel Test [91], [92]. We explored 88 model variants, including all combinations of substitution schemes, equal or unequal base frequencies, a proportion of invariant sites, and gamma distributed rate variation. The Akaike Information and Bayesian Information Criteria (AIC and BIC respectively) were examined to determine the optimal models. Both tests agreed on the Tamura Nei (TN) model with Invariants estimated and a Gamma distribution (TN+IG), as well as a General Time Reversible (GTR) model with only invariant sites included (GTR+I), as being the optimal models with equivalent information content.

For both the nucleotide and amino acid phylogenies, a ML tree was constructed using the program PhyML [91] and run with both the TN+IG model and more complicated GTR+I model. Since both reconstructions were nearly identical, all subsequent analyses were performed using the TN+IG model. The branch lengths were estimated using maximum likelihood, and the branch supports were created two ways. First, PhyML was run in a bootstrapping framework and replicated 100 times. The set of trees created was used to create a consensus tree, and the percentage of times that the clade appeared was calculated. In addition, the approximate likelihood ratio statistic (aLRS) was calculated and transformed directly to the Likelihood Ratio. Lastly, we used a Bayesian approach to reconstruct the phylogeny, including estimates of the branch length and support for branches in terms of posterior probabilities. Bootstrap, Likelihood Ratios and Bayesian Posterior Probabilities are presented on the branch lengths of the phylogenies.
